# Highly Responsive Ultraviolet Sensor Based on ZnS Quantum Dot Solid with Enhanced Photocurrent

**DOI:** 10.1038/s41598-019-55097-8

**Published:** 2019-12-10

**Authors:** Sellan Premkumar, Devaraj Nataraj, Ganapathi Bharathi, Subramaniam Ramya, T. Daniel Thangadurai

**Affiliations:** 1 Quantum Materials and Devices Laboratory, Department of Physics, Bharathiar University, Coimbatore, Tamil Nadu 641046 India; 2 UGC-CPEPA Centre for Advanced Studies in Physics for the development of Solar Energy Materials and Devices, Department of Physics, Bharathiar University, Coimbatore, Tamil Nadu 641046 India; 3 School of Chemistry and Chemical Engineering, Tianjin Polytechnic University, Tianjin, 300387 China; 4 Tianjin Key Laboratory of Green Chemistry and Process Engineering, and School of Material Science and Engineering, Tianjin Polytechnic University, Tianjin, 300387 China; 5 Department of Nanoscience and Technology, Sri Ramakrishna Engineering College, Coimbatore, Tamil Nadu 641022 India

**Keywords:** Optical properties and devices, Quantum dots

## Abstract

Detection of visible blind UV radiation is not only interesting but also of technologically important. Herein, we demonstrate the efficient detection of UV radiation by using cluster like ZnS quantum dot solid nanostructures prepared by simple reflux condensation technique. The short-chain ligand 3-mercaptopropionic acid (MPA) involved in the synthesis lead to the cluster like formation of ZnS quantum dots into solids upon prolonged synthesis conditions. The ZnS QD solid formation resulted in the strong delocalization of electronic wave function between the neighboring quantum dots. It increases the photocurrent value, which can be further confirmed by the decrease in the average lifetime values from 64 to 4.6 ns upon ZnS cluster like QD solid formation from ZnS QDs. The ZnS quantum dot solid based UV sensor shows good photocurrent response and a maximum responsivity of 0.31 (A/W) at a wavelength of 390 nm, is not only competitive when compared with previous reports but also better than ZnS and metal oxide-based photodetectors. The device exhibits a high current value under low-intensity UV light source and an on/off ratio of I_UV_/I_dark_ = 413 at zero biasing voltage with a fast response. Further, photocurrent device has been constructed using ZnS quantum dot solid nanostructures with graphene hybrids as an active layer to improve the enhancement of photoresponsivity.

## Introduction

UV-photodetectors received great scientific attention owing to their significant commercial applications including water treatment, defense safety^1^, flame detection, and space communication^2–5^. The commercially used silicon-based photodetector faces a major problem of low photon absorption capability in the Ultraviolet (UV) region due to its high reflection co-efficient^6^. Recently, numerous technological innovations have been reported in the detection of UV radiation, where several wide bandgap semiconducting materials were used for the photoactive materials in UV-photodetectors namely, SiC^6,7^, ZnS^8^, GaN^9–11^, ZnO^12–14^, Ga_2_O_3_^7,15,16^ and Graphene QDs^17–19^. UV Photodetector constructed by using Metal oxide semiconductor as an active material delivers poor device performance due to its surface defects^20,21^, which causes the slow recovery of photocurrent. Metal chalcogenides (S, Se, Te)^5,22–24^ based photodetectors were considered to be the best alternative to metal oxide based photodetectors. The photoconductive type^8^ and photodiode type technologies were mainly used in current research and commercial products of the UV photo-sensing field. Having a wide band gap semiconductor is a potential considerate for constructing UV photodetectors. There are several reports on the investigation of ZnS nanostructure based UV photodetectors^25–27^. For example; Yeonhokim *et al*.^28^ demonstrated 1D ZnS nanobelt with graphene structure producing the responsivity of 1.2 mW/cm^2^ power when illuminated at 300 nm UV light, where it delivered the photocurrent value of 0.115 mA^29^. Fang *et al*., has reported ZnS nanobelts based photodetectors with the photocurrent value of 0.5 pA resulting in the low responsivity of 0.1 A/W^23^. An Qinwei et al also reported ZnS nanotube with silver nanowire-based UV photodetector^27^. However, these reported photodetectors displayed relatively low responsivity and slow photoresponse, caused by low carrier mobility. The three-dimensionally confined ZnS quantum dot system has been investigated over other nanostructures, because of its superior quality: higher surface to volume ratio. But it has some difficulties in the electron transfer process between the quantum dots. The colloidal quantum dots are able to generate the electron-hole (e-h) pairs effectively; however all the photogenerated charges could not be transfered to the respesective electrodes to yield the best device performance. The photogenerated charges have to travel through the adjacent quantum dots, where they get trapped before reaching the electrodes. In addition, the large capping molecules will offer resistance to the transfer of charges between quantum dots.

In recent years a number of innovative research work were undergoing in the construction of ZnS nanostructure^29–31^ based UV-photodetector with different morphologies like ZnS nanobelts^8,23,28,31,32^, ZnS nanotubes^27^ and ZnS nanowires^33,34^, as an active layer. These type of one-dimensional^35^ nanostructure-based UV photodetectors show relatively low responsivity and slow photoresponse due to low carrier mobility and high surface reactivity. ZnS Quantum dot has poor charge transport issue; as discussed before, in QDs system ligands molecules negative influence on the charge transport properties^36–38^. One possible way to address this problem is bringing the QDs closer enough by means of using short-chain ligands over the widely used lengthier ones (such as TOPO, TOP, Oleic acid, Oleylamine)^37,39,40^. Interconnecting the quantum dots with the help of short-chain ligands should enhance the performance of the device owing to its excellent inter-dot charge transport^41–44^. Such type of nanostructures is normally denoted as Quantum dot solids as it mimics the character of atomic solids. Like the arrangement of individual atoms can lead to the formation of atomic solids, quantum dot solid can be formed by means of an effective arrangement of quantum dots. In our previous work, we have attained CdTe QDs solid wire-like structure in the prolonged reaction condition, which shows better photocurrent response than quantum dots based device^43^. The bulk bandgap of CdTe lying in the visible region makes it more suitable for solar cell applications than acting as an UV sensing device. ZnS nanostructures have the bulk bandgap in the UV region making it a perfect choice as an active layer for UV photo-sensing device. With this basic knowledge, we have tuned the morphology of ZnS quantum dots to cluster like ZnS QDs solids by simply prolonging the reaction time. UV photodetector constructed using clusters like ZnS QDs solid exhibited better device performance over ZnS quantum dot based device due to the effective overlapping of electronic wavefunctions of neighboring quantum dots in the cluster state. To the best of our knowledge, these kinds of an interesting cluster like ZnS quantum-dot based photodetector device is not yet constructed till now. For attaining the improved charge carrier extraction and responsivity further, the ZnS quantum dot cluster like nanostructures were hybridized with graphene and used as an active layer for photodetector device^45,46^. The present paper discusses the methodology used for attaining clusters like nanostructures and their characterization, then finally the photocurrent study under UV light illumination.

## Results and Discussion

### Cluster like ZnS quantum dot solid formation

**Figure 1:**
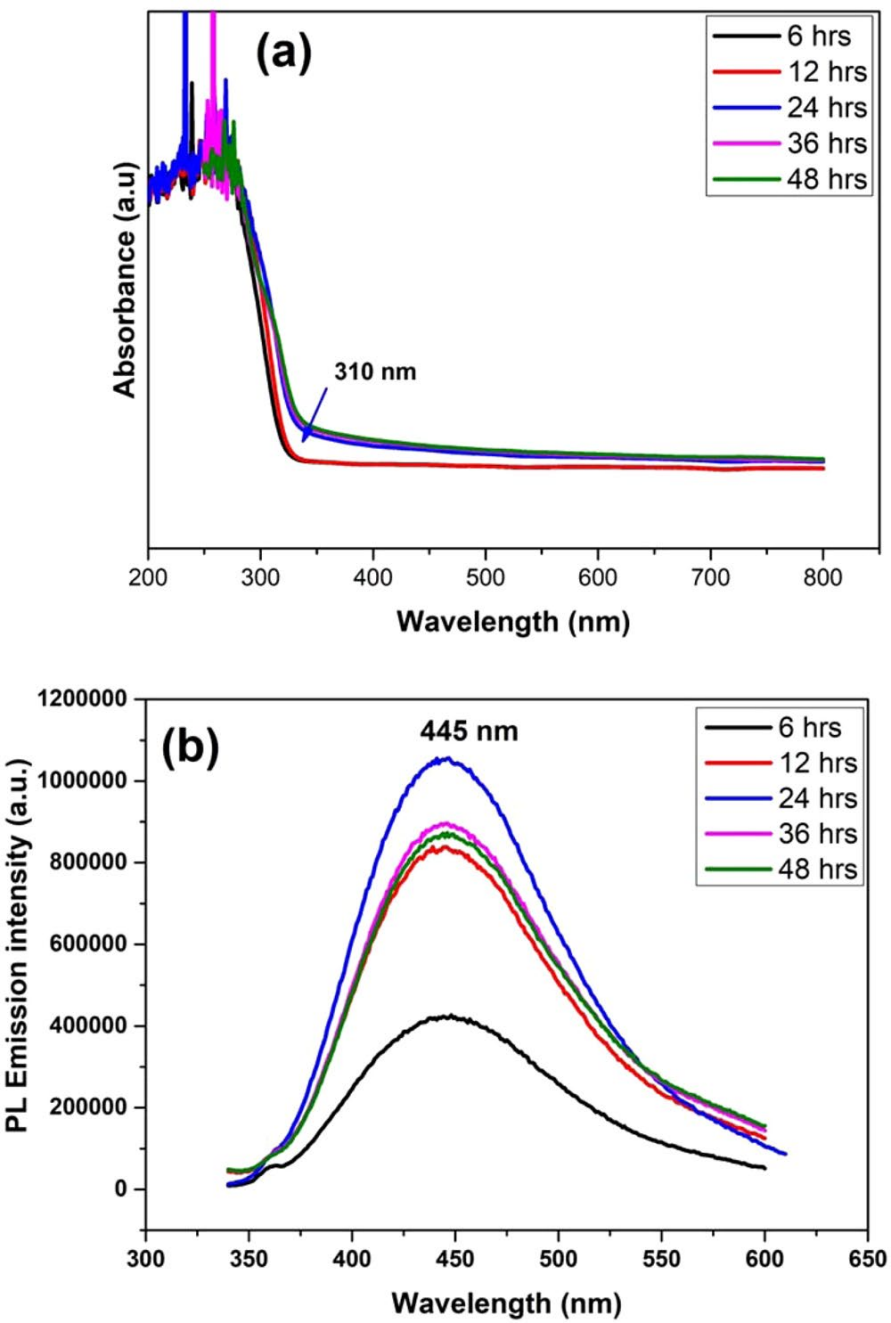
(**a**) UV-vis Absorbance spectra, and (**b**) Photoluminescence emission spectra of ZnS QDs samples prepared at different reaction time intervals (6, 12, 24,36, and 48 hrs).

Figure 1(a,b) shows the UV-Vis absorption and PL emission spectra of ZnS samples prepared at different reaction time intervals. The optical UV absorption edge of the ZnS 6 hrs sample is observed around at 310 nm with a significant blue shift compared to the bulk ZnS band gap value of 340 nm^47^ owing to the quantum confinement effect. To know about the emission behavior of these samples, we have recorded photoluminescence emission spectra by exciting the samples at their respective absorption maximum. Upon excitation at 310 nm the ZnS quantum dot exhibit emission around 445 nm which is shown in Fig. 1(b). The transition of electrons from a shallow state near the conduction band to the sulfur vacancies present near the valence band is the major reason for the emission observed at 445 nm (Fig. S1)^48^. Upon increasing the reaction time from 6 hrs we have noticed the improvement in the intensity of emission peak up to 24 hrs. Once when the reaction time crosses 24 hrs, the emission peak intensity gets decreased. This observation indicates the possibility of changes in the morphology of the samples, which were subjected to the prolonged synthesis duration. In order to quantify the morphological change, the samples are subjected to HR-TEM analysis with respect to the reaction time.

**Figure 2:**
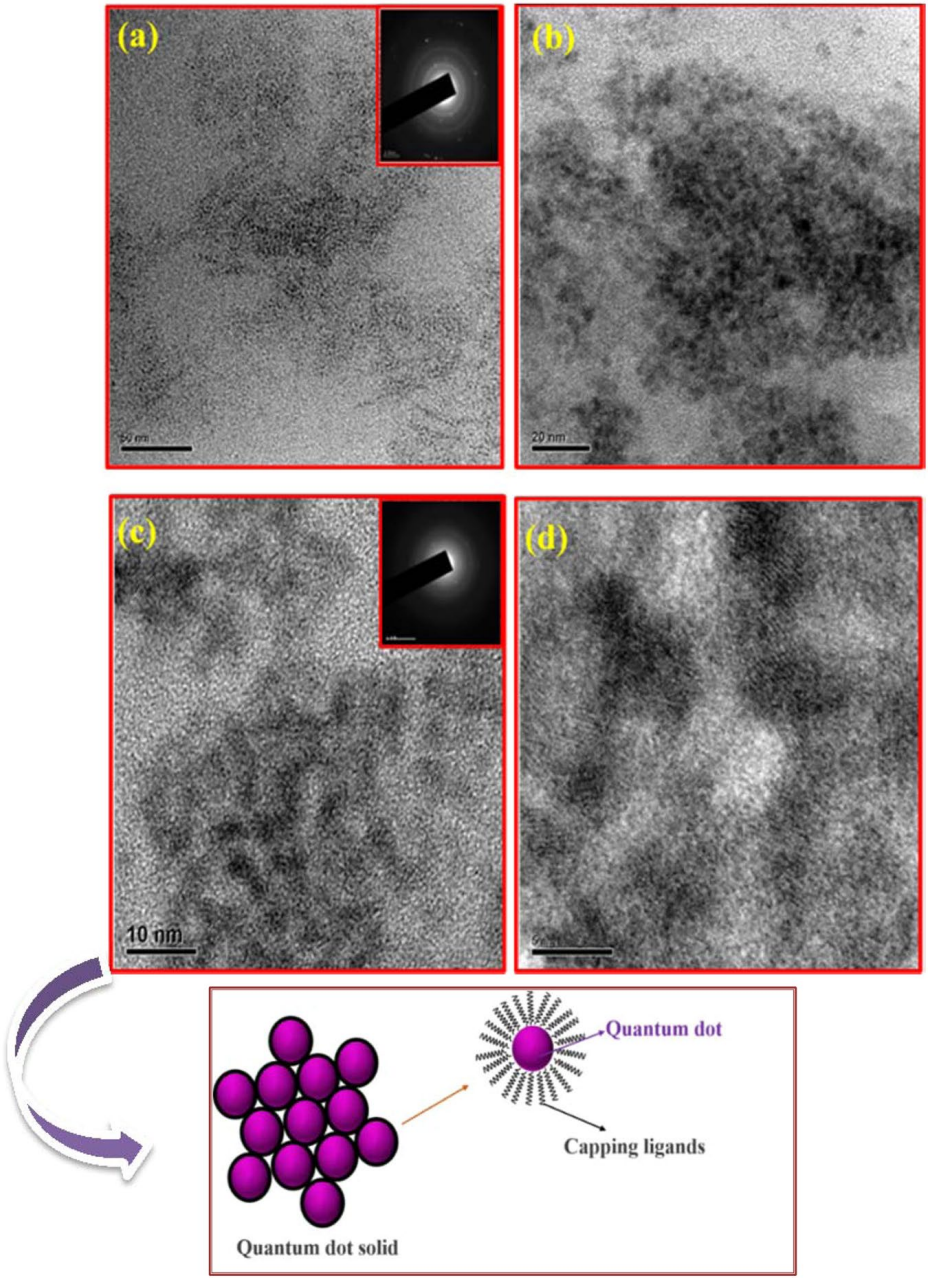
HRTEM analysis of ZnS QDs samples prepared at different reaction time intervals (**a**,**b**) ZnS QD (12 hrs) (**c**,**d**) ZnS QD solid (24 hrs) (inset is SAED pattern of ZnS). Schematic representation of Quantum dot solids (Down).

**Figure 3:**
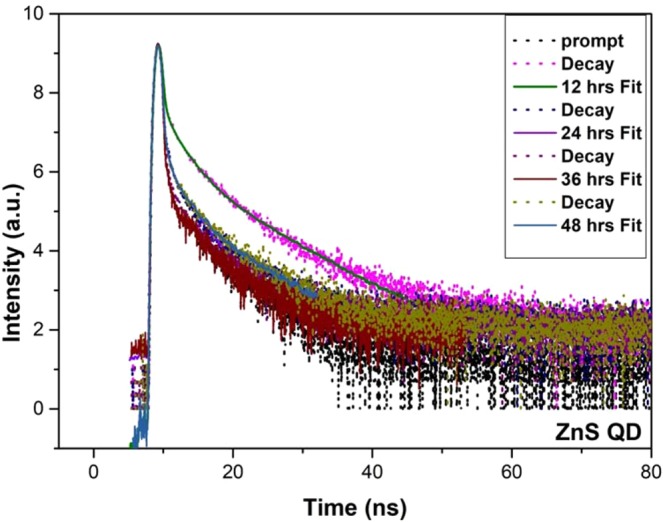
TCSPC lifetime spectra for Pure ZnS QDs prepared at different reaction time intervals (12, 24, 36 and 48 hrs).

Figure 2(a–d) shows the HR-TEM results of ZnS quantum dot samples prepared at different reaction time duration such as 12 hrs and 24 hrs. Having a closer look at the HR-TEM images as shown in Fig. 2(a,b), one can see that there was no increase in the size of ZnS QDs beyond 5 nm; instead the ZnS QDs starts to agglomerate and form clusters with an increase of reaction time after 24 hrs. It means that the ZnS QD joins together to form a quantum dot cluster like solid structure. In this cluster state, the electronic wavefunctions of neighboring quantum dot get overlapped which leads to better carrier transport because of the delocalization effect. Due to this delocalization effect, we have attained quenching in emission starting from 24 hrs. The final hour reaction sample exhibits large clusters that contain interconnected ZnS quantum dots as shown in Fig. S2(a,b). The 48 hr prepared samples in the HRTEM images shows large size quantum dots clusters formed by the joining of individual quantum dots during prolonged time synthesis. We have subjected the as-prepared ZnS samples to TCSPC analysis by using a 390 nm pulsed excitation source. The PL decay lifetime curve is fitted with bi-exponential. It shows that the decay time of ZnS QDs decreased gradually with increasing reaction time intervals from 12 to 48 hrs as shown in Fig. 3. The calculated lifetime values presented in the Table 1, represents the decreases in the average lifetime values from 64 to 4.6 ns upon ZnS cluster like QD solid formation from ZnS QDs. It has to be noted that the faster component life time (τ_1_) has an increased contribution to the overall lifetime value from 46% to 90%. From the above observation, we concluded that the cluster like ZnS quantum dot solid formation has resulted in the delocalization of excited charge carriers into nearby ZnS QDs, in the cluster like quantum dot solid structure, which decreases the PL emission intensity and corresponding reductions in the average lifetime. We believe that these interesting results could be effectively utilized to harvest the photoelectrons with enhanced performance in the photodetector application. Precisely, when the excitons are delocalized in a coupled QD solid system, their exciton binding energy decreases. This expected decrease in the binding energy charge carrier will make them available for relatively longer time at the excited states of the coupled system, and can be collected easily using appropriate electrodes. By collecting these delocalized excitons, the resultant photocurrent can be improved. The reported inorganic semiconductor-based UV photodetectors still have low electrical performance (charge transport properties). To overcome this limitation, the widely used option is hybridization with graphene, a better alternative to increase the photocurrent efficiency and the overall device performances. Herein, the graphene hybridization protocol is also utilized to extract the photoexcited electrons from the ZnS QDs nanostructure.

**Table 1:** TCSPC lifetime values for Pure ZnS quantum dot samples at different reaction time intervals.

Reaction Time	Lifetime [τ_1_]	Lifetime [τ_2_]	Avg. Lifetime [τ]
ZnS 12 hrs	τ_1_ = 3.004 × 10^−10^s (*A*_1_ = *46.7*2)	τ_2_ = 6.44 × 10^−8^s *(A*_*2*_ = *53.28)*	τ = 64 × 10^−9^s
ZnS 24 hrs	τ_1_ = 1.4355 × 10^−10^s (*A*_1_ = *90.70*)	τ_2_ = 5.730 × 10^−9^s *(A*_2_ = *9.30)*	τ = 4.636 × 10^−9^s
ZnS 36 hrs	τ_1_ = 1.386 × 10^−10^s *(A*_1_ = *87.9*2*)*	τ_2_ = 5.9980 × 10^−9^s *(A*_*2*_ = *12.08)*	τ = 5.15 × 10^−9^s
ZnS 48 hrs	τ_1_ = 1.4739 × 10^−10^s *(A*_1_ = *83.81)*	τ_2_ = 5.2789 × 10^−9^s (*A*_2_ = *16.19*)	τ = 4.63 × 10^−9^s

### Investigation of graphene/ZnS quantum dot hybrids structure

**Figure 4:**
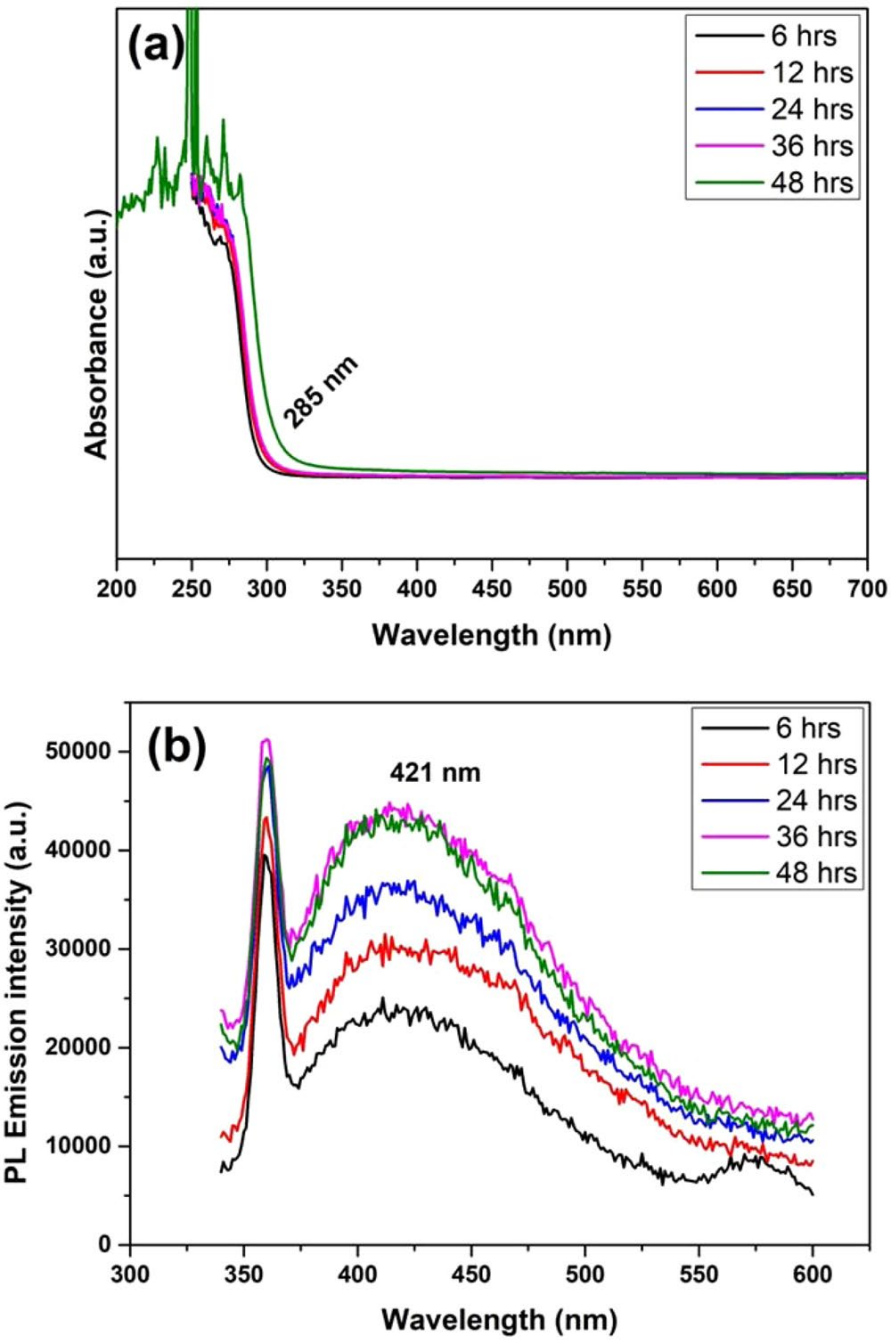
(**a**) UV-vis Absorbance and (**b**) Photoluminescence emission spectra of ZnS QDs with graphene hybrids samples prepared at different reaction time intervals.

**Figure 5:**
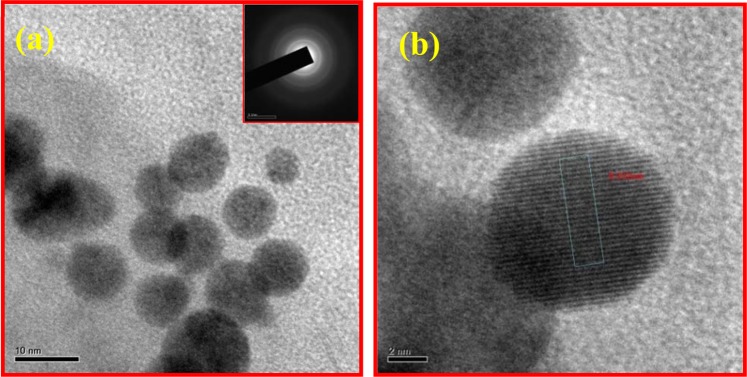
HRTEM images of ZnS QD with graphene hybrids samples: (**a**) Low resolution images of graphene sheets decorated ZnS QDs (24 hrs) and (**b**) High resolution image clearly showing the QD lattice d spacing value of ~0.22 nm with corresponding 111 planes of cubic ZnS.

From the literature reports, graphene has been widely used as the electron extractor or charge carrier collector to improve the efficiency of the optoelectronic devices^49–51^. In particular, the use of graphene to prepare efficient hybrid nanostructures in the field of solar cells and photodetectors has gained remarkable attention in recent years^17,29,51^. The reported hybrid nanostructures include Silicon/Graphene^6^, ZnS/graphene^28,29^, CdSe/graphene^52^, CdTe/graphene^53,54^, and ZnSe/ZnS:Graphene^30^. The responsivity of the photodetectors can be improved by effectively harvesting the excited charge carriers, irrespective of the illumination energy, i.e., the photoexcited charge carriers must be harvested from the photosensitizers before the recombination takes place. As discussed before, one possible way to achieve this is to harvest the excited charge carriers, immediately after the photoexcitation^55,56^. In the present case, we have introduced graphene in such a way that, it can be able to collect the photoexcited electrons from ZnS QDs, which in turn would enhance the photocurrent response of the photocurrent device. The graphene/ZnS quantum dot hybrids were synthesized by a modified protocol as described in the experimental section. Similar to the previous synthesis procedure, the samples were generated at different time intervals and then subjected to UV-vis absorption, PL emission and HRTEM analysis to investigate its morphological and optical properties. Figure 4(a,b) shows the optical UV absorption and PL emission spectra respectively, recorded for ZnS QD/graphene hybrids samples. In the case of 6 hrs sample, the blue-shifted absorption peak is observed at 285 nm compared to that of pure ZnS QDs sample, which confirms the formation of hybrid structures. The PL emission quenching was observed in the hybrid samples and weak blue-shifted emission at around 421 nm, compared to the bare ZnS samples (presented in Fig. 4(b)), which is recognized due to the transfer of photoexcited electrons from ZnS QDs to graphene system^57,58^. Further increasing the reaction time, increases the PL emission intensity up to 36 hrs, after which it remained the same. Figure 5(a,b) shows the monodispersed spherical shaped ZnS QDs particle decorated on the graphene layer (24 hrs) with an average particle size of about 8 nm, and the measured lattice d-spacing value corresponding to 111 planes of cubic ZnS is found to be 0.22 nm. The lattice fringes visible in the HRTEM confirm the crystalline nature of the ZnS QDs.

**Figure 6:**
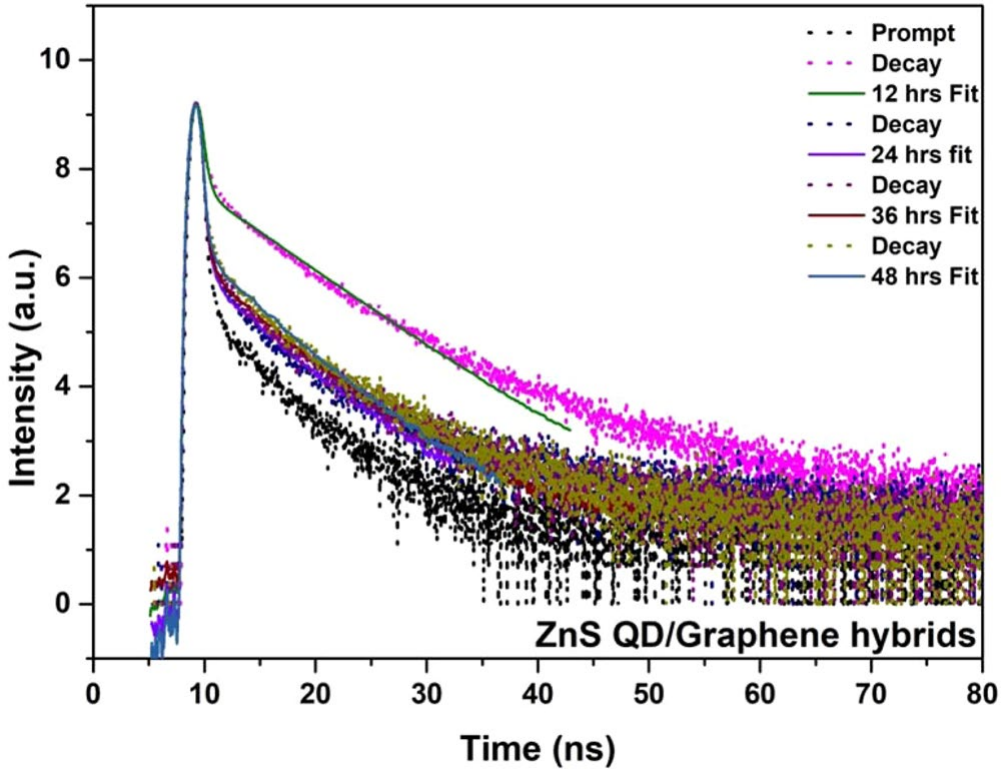
TCSPC lifetime spectra of ZnS/Graphene hybrids prepared at different reaction time intervals (12, 24, 36 and 48 hrs) respectively.

**Figure 7:**
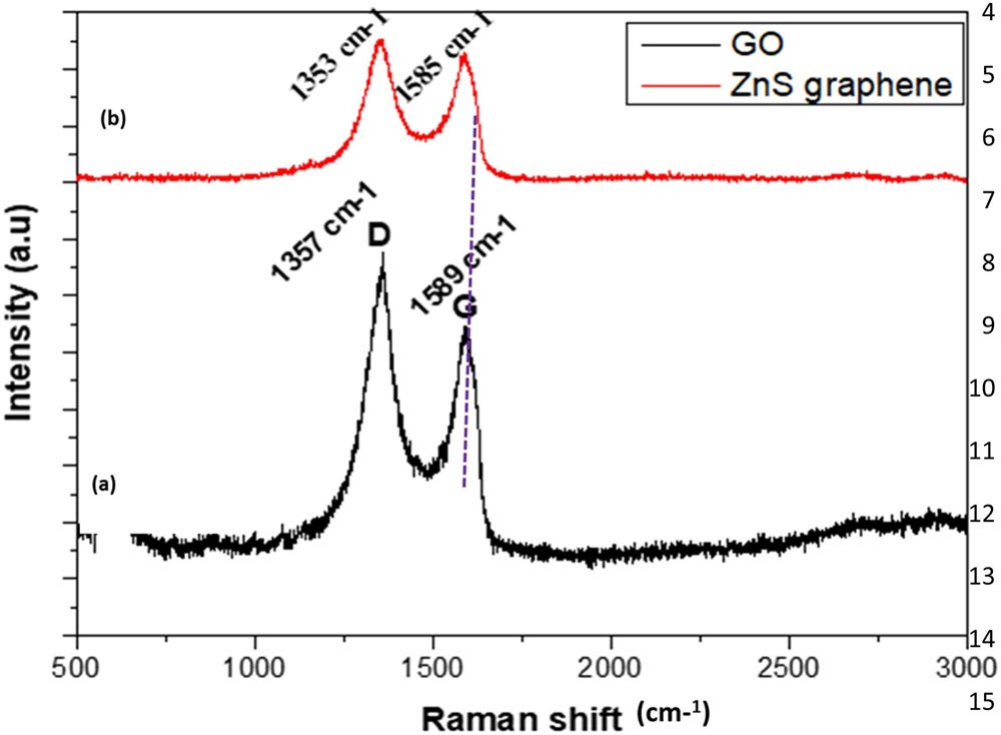
The Raman spectra of (**a**) GO, (**b**) ZnS/Graphene hybrids sample prepared at 24 hrs. Photocurrent measurement for ZnS quantum dot solid and its graphene hybrids.

In addition, the interaction of ZnS QDs with graphene was investigated by the PL decay lifetime by using TCSPC measurements. Figure 6 shows the PL decay profile of graphene/ZnS QDs samples. The average lifetime values were calculated and presented in Table 2, which indicates the decrease in average lifetime value from 43 ns to 3.7 ns with increasing reaction time from 12 hrs to 48 hrs. On comparing these results to that of bare ZnS QDs sample, it is clear that there must be some electronic interactions between the ZnS QDs and hybridizing graphene, which has resulted in the overall decrease of average lifetime values of hybrid samples^53,54^. The hybridization of ZnS QDs with graphene was further confirmed by the observed shift in D and G Raman bands. The graphene oxide and graphene/ZnS hybrids samples were subjected to Raman analysis and the obtained spectra are shown in Fig. 7(a,b). While two typical peaks of GO can be found at 1357 and 1859 cm^−1^, corresponding to D and G bands respectively, the hybrid samples exhibit D and G bands at 1353 and 1585 cm^−1^, respectively. The observed redshift in the Raman bands of hybrid samples, indicated the reduction of graphene oxide and the hybrid formation with ZnS QDs as reported elsewhere^46,59^. In the hybrids samples, the two peaks obtained at D and G bands indicates the sp^2^ carbon networks and the relative intensity ratio of the I_D_/I_G_ represents the degree of carbonization. Further, the redshift in the G band position was observed due to the softening of phonons, which indicated the enrichment of electrons in graphene. i.e., the electrons are transferred from ZnS QDs to graphene upon hybrid formation as reported elsewhere^60^. Further, we confirmed the graphene hybrids formation by using FTIR spectra results as shown in Fig. S3.

**Table 2:** TCSPC lifetime values of ZnS QD/Graphene hybrids prepared at different reaction time intervals.

Reaction Time	Lifetime [τ_1_]	Lifetime [τ_2_]	Avg. Lifetime [τ]
ZnS/Graphene12 hrs	τ_1_ = 2.2643 × 10^−9^s (*A*_*1*_ = *13.38)*	τ_2_ = 4.40 × 10^−8^s *(A*_2_ = *86.62)*	τ = 43.67 × 10^−9^s
ZnS/Graphene 24 hrs	τ_1_ = 1.2490 × 10^−10^s *(A*_1_ = *91.65)*	τ_2_ = 2.675 × 10^−9^s *(A*_2_ = *8*.3*5)*	τ = 1.810 × 10^−9^s
ZnS /Graphene 36 hrs	τ_1_ = 1.9290 × 10^−9^s *(A*_1_ = *3*.2*0)*	τ_2_ = 4.218 × 10^−8^s *(A*_*2*_ = *3.90)*	τ = 6.10 × 10^−9^s
		τ_3_ = 9.8339 × 10^−11^s (*A*_*3*_ = *92.90*)	
ZnS/Graphene 48 hrs	τ_1_ = 4.801 × 10^−9^s *(A*_*1*_ = *8.70)*	τ_2_ = 1.2813 × 10^−10^s *(A*_*2*_ = *91.30)*	τ = 3.786 × 10^−9^s

### Photocurrent measurement for ZnS quantum dot solid and its graphene hybrids

**Figure 8:**
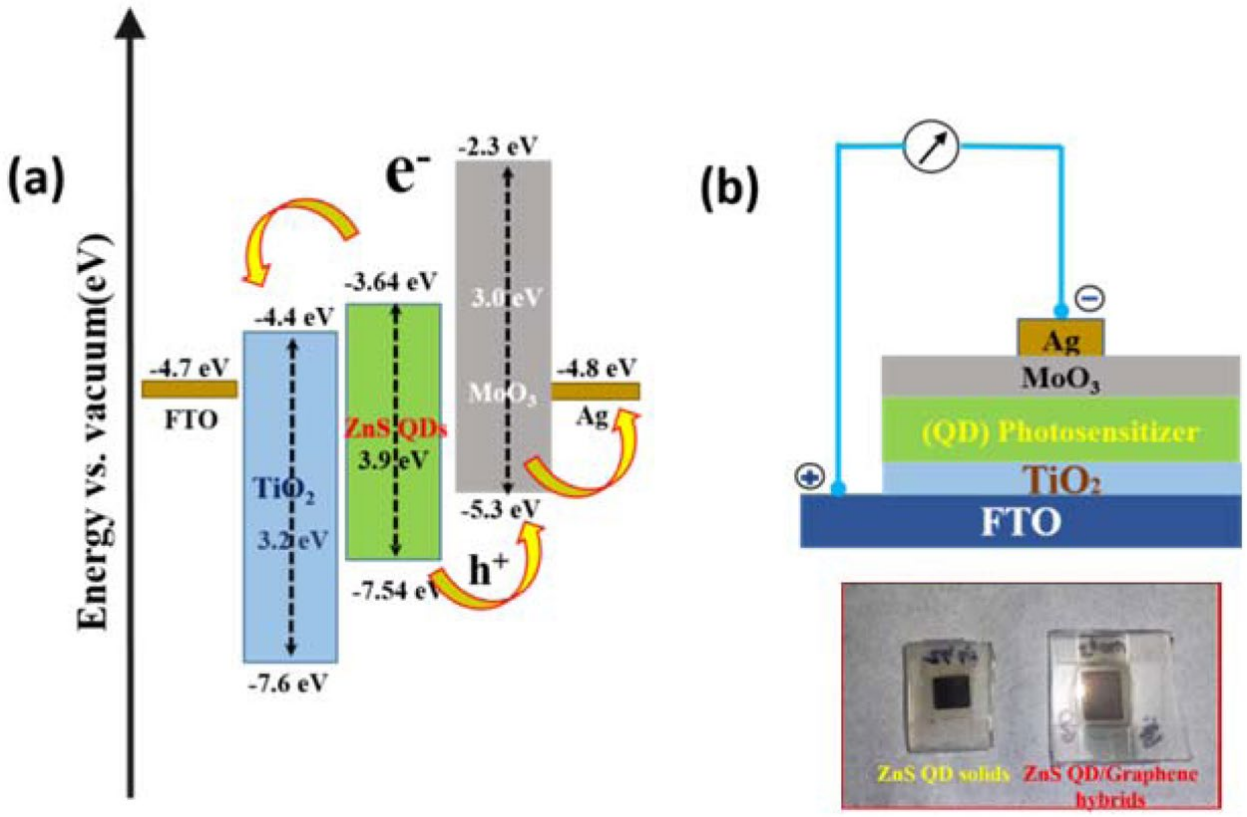
(**a**) Schematic representation of relative energy level position for ZnS QDs solid and (**b**) Photocurrent device structure.

**Figure 9:**
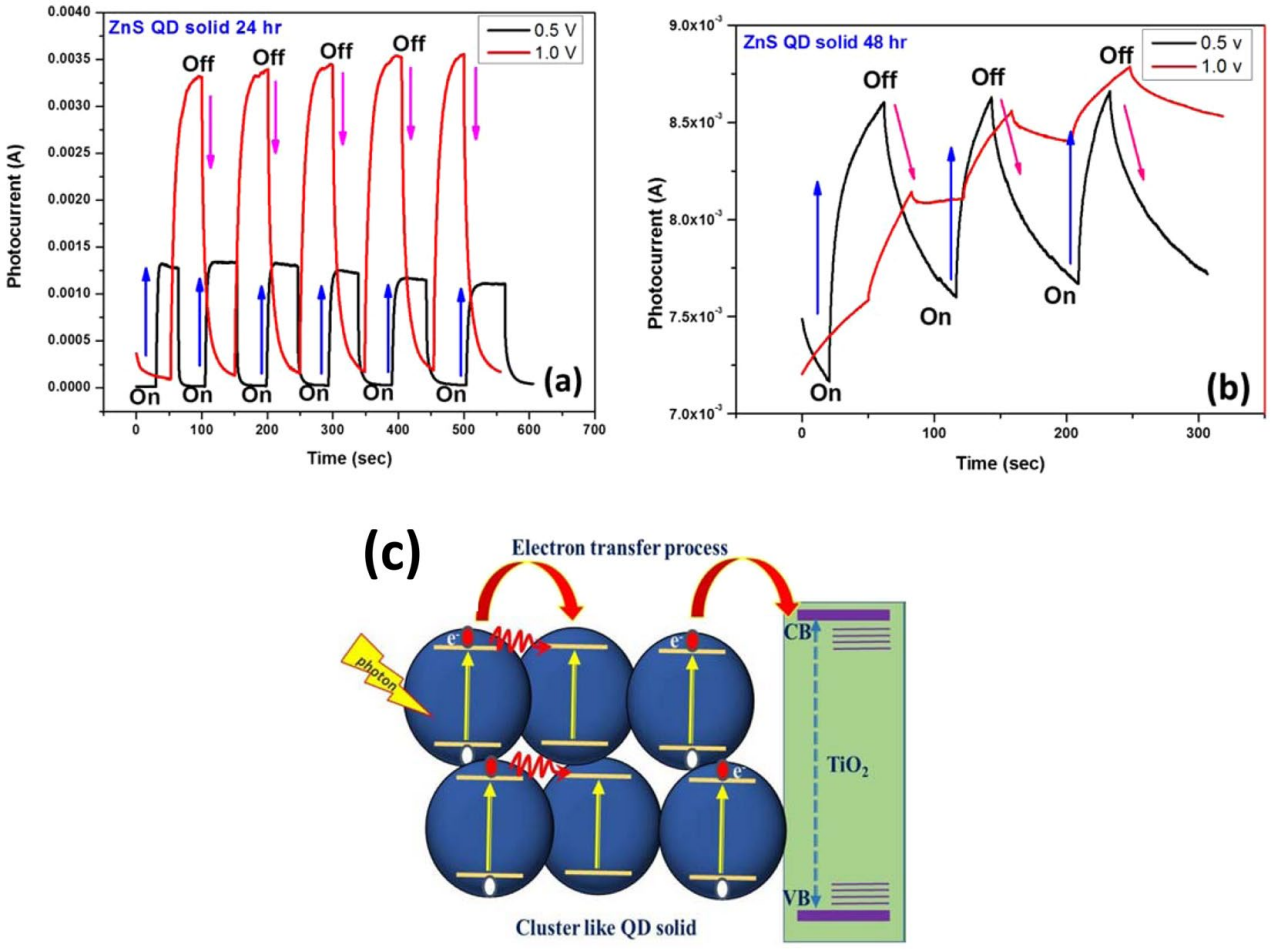
Photocurrent properties for ZnS QD solid prepared at (**a**) 24 hrs, (**b**) 48 hrs, samples recorded at different biasing voltage (0.5 and 1.0)Volt and (**c**) Schematic representation of electron transfer process between the cluster like QD solid and TiO_2_ film reducing the electron recombination with increasing electron transfer rate.

**Figure 10:**
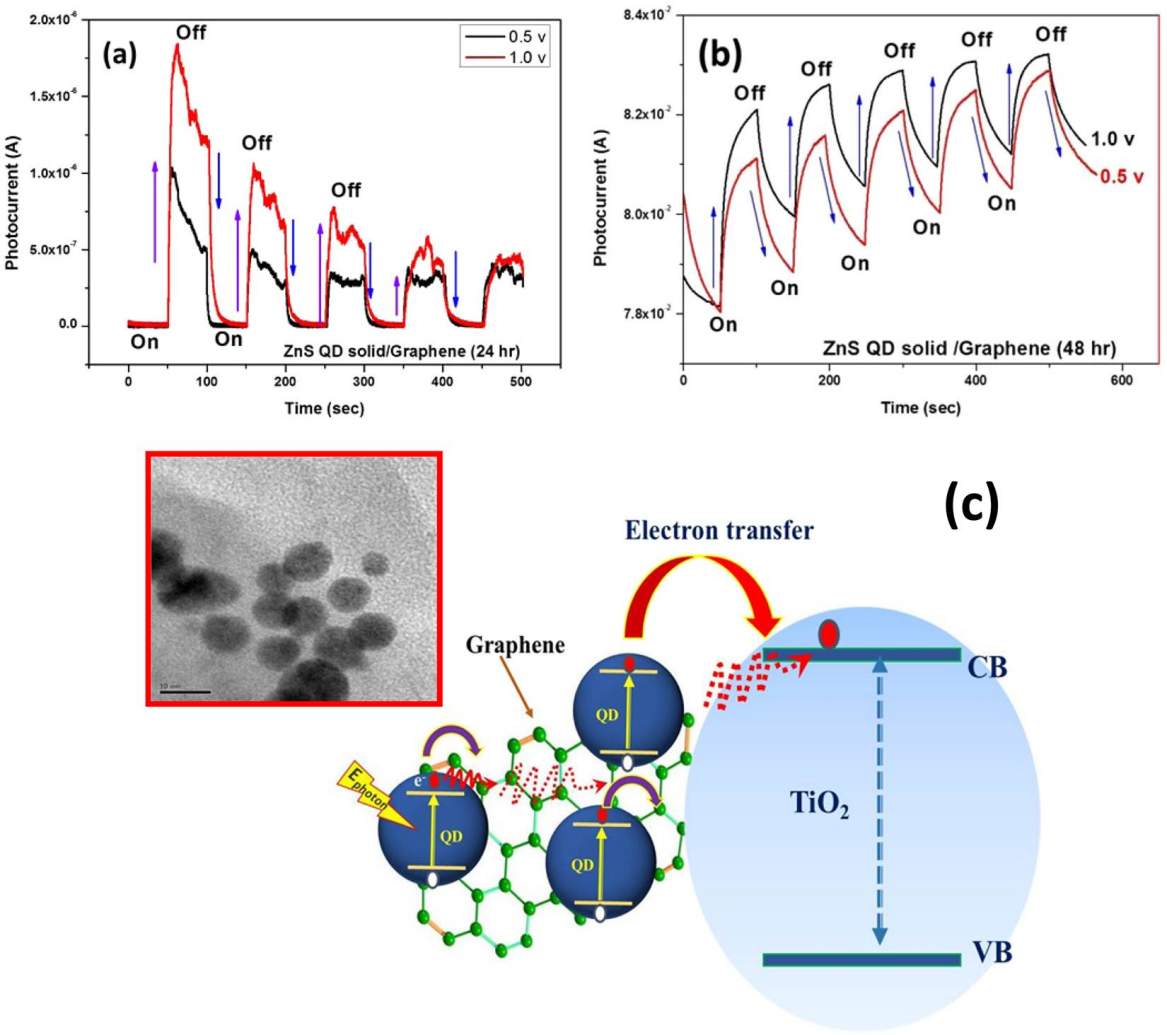
Photocurrent properties for ZnS QD with graphene hybrids (**a**) ZnS QD solid with graphene prepared at 24 hrs (**b**) ZnS QD solid with graphene prepared at 48 hrs, samples recorded at different biasing voltage (0.5 and 1.0)Volt, and (**c**) Schematic representation of electron transfer from QDs/graphene hybrids to TiO_2_.

The photocurrent characterization of ZnS clusters like quantum dot solid and ZnS QD decorated graphene hybrids were investigated by incorporating them as the active layer in the (FTO/TiO_2_/(photosensitizer)/MoO_3_/Ag) device structure as shown in Fig. 8; here the photo-sensitizer denotes the as-prepared cluster like ZnS quantum dot solid and ZnS/Graphene hybrid samples (both 24 hrs and 48 hrs samples). The photoresponse curves recorded for the biasing voltages of 0.5 V and 1.0 V are presented in Figs. 9 and 10, respectively. From the photocurrent curves, it is clear that all the samples show higher photocurrent value for the biasing voltage of 1.0 V (compared to the corresponding values measured with 0.5 V biasing voltage). The ZnS QD solid (24 hrs prepared sample), generated the maximum photocurrent values of 1.0 mA and 3.5 mA for the biasing voltages of 0.5 V and 1.0 V as shown in Fig. 9(a). Surprisingly, the ZnS QD solid samples collected at 48 hrs reaction, displayed a sharp rise in the photocurrent to 8.6 mA (for the biasing voltage of 1.0 V), however with a low on/off ratio (I_on_/I_off_ = 1.2) compared with 24 hrs sample, which displayed a better ON-Off ratio of 1.58 × 10^2^ along with a fast response time of <0.05 sec as shown in Fig. 9(a,b). The higher photocurrent value observed in the case of ZnS QD solid samples prepared at 48 hrs reaction time attribute to the QD solid formation as explained in the previously. The observed non-zero dark current could be due to the leakage of charge carriers across the junction in the absence of illumination as reported elsewhere^61,62^. The schematic representation of the electron transfer process is shown in Fig. 9c. Figure 10(a,b) shows the photocurrent response of ZnS QD solid/graphene hybrids 24 hrs and 48 hrs samples, respectively, which indicate the maximum photocurrent values of 1.8 μA and 82 mA, respectively, for a biasing voltage of 1.0 V. The difference in the device performance when compared to 24 hrs ZnS QDs solid/graphene hybrid device can be explained as follows. For the preparation of ZnS QDs solid/graphene hybrid sample, graphene oxide has been used as a precursor. During the hybrid growth process, the GO was reduced as rGO by the hydrazine hydrate, a reducing agent added during the reaction. This reduction of GO further continues and when the reaction time reaches 48 hrs time, the rGO further reduced and etched into graphene quantum dots (GQDs)^43^. We believe that the formation of GQDs contribute the resultant photocurrent and it is because of this reason, the ZnS QD solid/graphene hybrid with 48 hrs prepared device a generated higher photocurrent value of 82 mA. Also, we believe that, in the 48 hrs ZnS QDs solid/graphene hybrids sample, the QD solid formation is nearly complete and therefore it appears like the aggregated QDs.

### Ultraviolet (UV) sensor device fabrication

**Figure 11:**
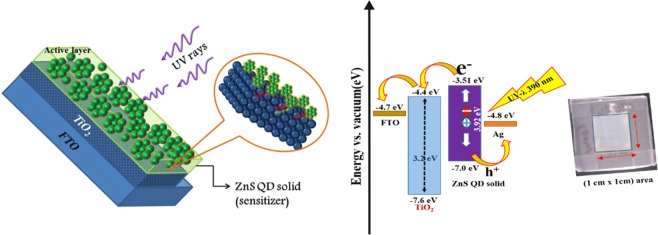
Schematic representation of relative energy level position for ZnS QDs solid (left), UV photo detector based device structure (right).

**Figure 12:**
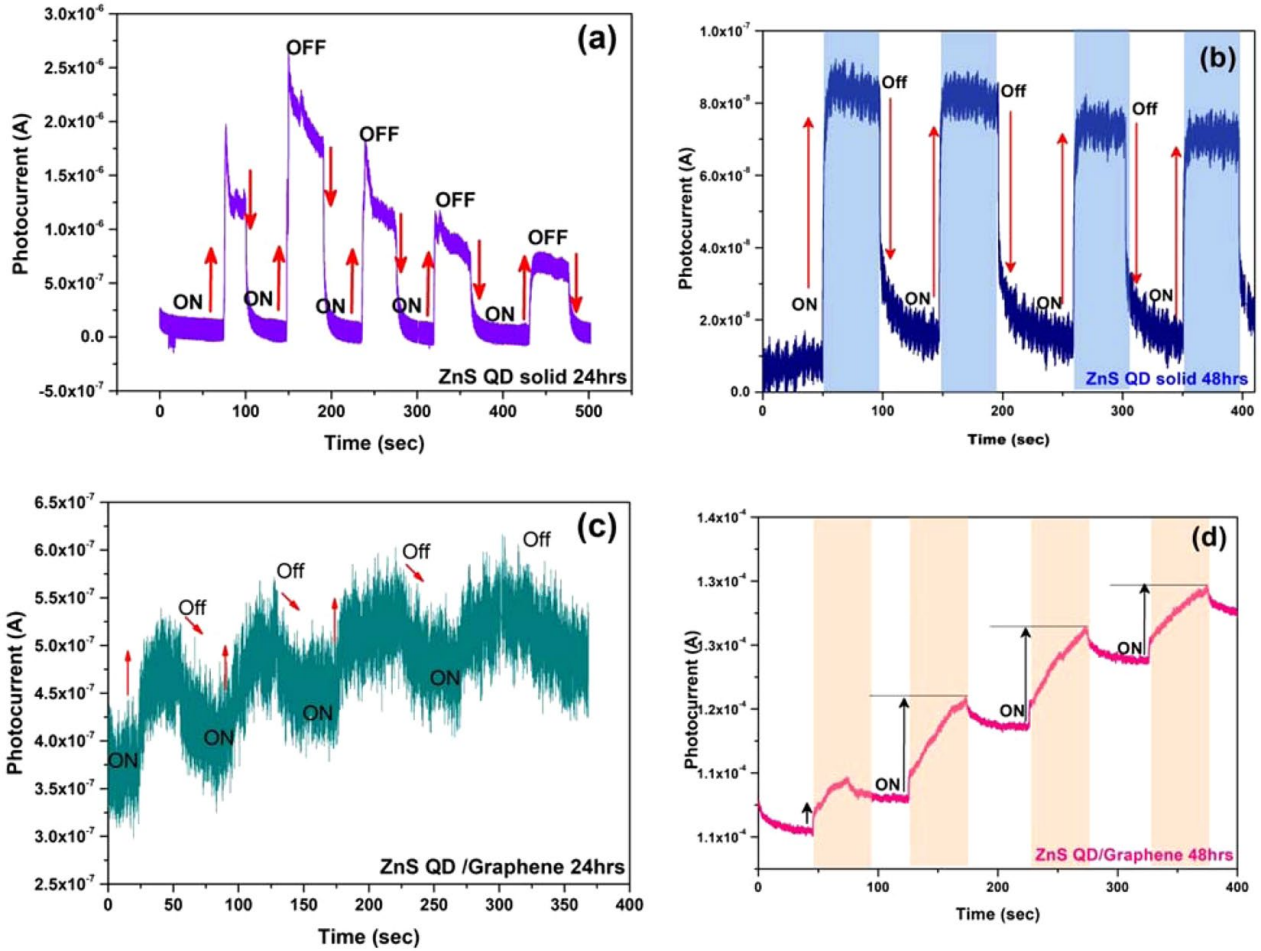
Time-dependent UV response behaviour, measured with turning 390 nm A UV light of 8 µW Cm^2^ on and off periodically in ambient condition. (**a**) ZnS QD solid 24 hr, (b) ZnS QD solid 48 hr, (**c**) ZnS QD solid/Graphene hybrid 24 hr and (**d**) ZnS QD solid/Graphene hybrid 48 hr (illumination of UV light ~390 nm).

**Figure 13:**
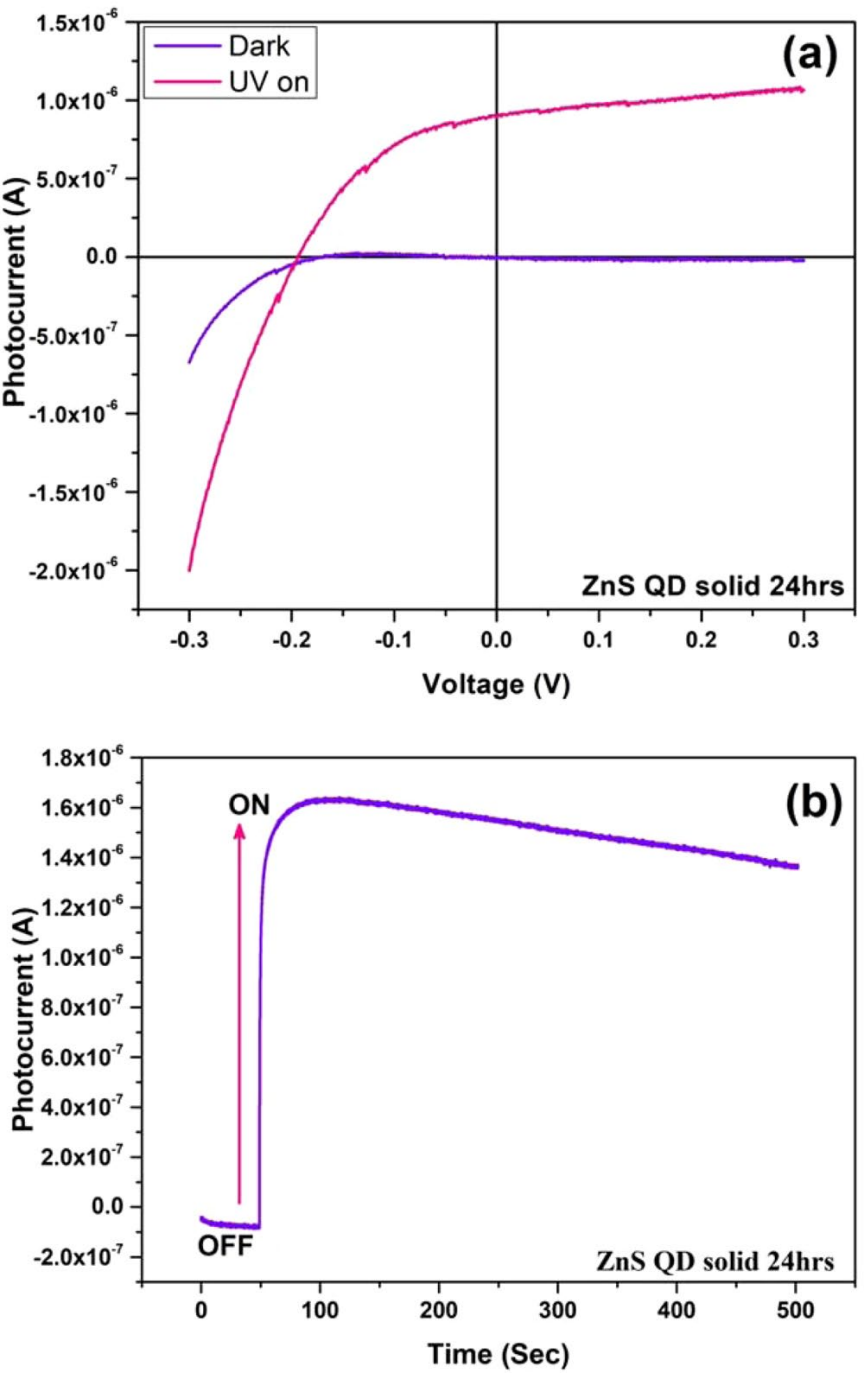
(**a**) I-V Characteristic of a ZnS cluster like QD solid 24 hrs sample based photodetector device under dark condition and UV light (**b**) and Stability curve under continuousUV light (390 nm) illumination.

The prototype UV sensor devices were fabricated using the as-prepared cluster like ZnS quantum dot solid and ZnS QD solid/Graphene hybrid samples (24 hrs and 48 hrs). The photocurrent device structure was FTO/TiO_2_/(active layer)/Ag. The as-prepared ZnS quantum dot solid and ZnS QD/Graphene hybrid samples were coated as the active layer, over the FTO/TiO_2_ and finally, the silver electrode was deposited as the top layer using the thermal evaporation technique as shown in Fig. 11. Figure 12 shows the recorded UV photocurrent response curves of the constructed photodetector devices. The photodetector devices constructed using ZnS quantum dot solid 24 hrs and 48 hrs samples displayed the maximum photocurrent values of 2.5 μA and 90 nA, respectively, at zero biasing voltage. On the other hand, the devices constructed using ZnS QD solid/Graphene hybrid samples prepared for 24 hrs and 48 hrs, generated the maximum photocurrent values of 0.6 μA and 0.11 mA, respectively (Fig. 12). The higher photocurrent observed in the case of ZnS QD solid/Graphene hybrid is attributed to the incorporation of graphene. The unrelaxed electrons present in the molecular energy levels of graphene add up to the photocurrent as the device ON time progresses. It is because of this reason, the saw tooth wave like pattern is observed for the ZnS QD solid/Graphene hybrid photodetector device. The maximum responsivity of this photodetector device is calculated to be 0.31 (A/W). It is worth noting that, the peak photocurrent of our best device (constructed using ZnS quantum dot solid 24 hrs sample) is much higher compared to the recently reported values^63,64^. Figure 13(a) shows the I-V characteristic curve under Dark and light illumination conditions. The stability curves are included in the Supporting Information (Fig. S4).

## Discussion

In order to clearly explain the working principle of the UV photodetector device, a simple energy band diagram is schematically presented in Fig. 11. Upon illumination (UV radiation), the device has created a number of photoexcited electrons in the sensitizer (ZnS QD solid), these excited state electrons move from the conduction band of ZnS_**CB**_ to TiO_2**CB**_^65,66^. And then the electrons are transferred to FTO (transparent conducting oxide) at the same time holes were collected from Ag in the favorable energy level position. Generally, quantum dot solid system having high electron mobility^41,42^ compared with the normal system, herein, ZnS quantum dots solid system have high electron mobility and delocalized electrons which has resulted in increasing the exciton generation (electron-hole pair) and the corresponding photocurrent as well. The observed photocurrent response under UV light illumination is attributed to the effective collection of photogenerated carriers from the photosensitizer, and the photodetector device shows higher sensitivity towards UV detection. The device exhibited a high magnitude current value under low-intensity UV light source and has the on/off ratio of I _UV_/I_dark_ = 413 at zero biasing voltage along with fast response and recovery time. The best result of our constructed photodetectors exhibited the maximum responsivity of 0.31 (A/W) with a photocurrent value of 2.5 µA at wavelength of λ = 390 nm.

In summary, the ZnS cluster like quantum dot solid system and ZnS QD decorated Graphene sheet hybrids were successfully prepared and their photophysical and UV photo-sensing properties were investigated in detail. The photophysical characterization of the bare ZnS system tipoff the enhancement in the interdot charge transport upon QD solid formation and provided the evidence for the photoexcited electron transfer from ZnS QDs to graphene in the hybrid system. These results were reflected in the device performances, by showing an enhancement in the photocurrent values of the respective UV sensor devices. Based on the overall device performances, the device constructed using ZnS QD solid 24 hr sample is better suitable for the practical application. Our best UV sensor device exhibited the maximum responsivity of 0.31 (A/W) with a photocurrent value of 2.5 µA at wavelength of λ = 390 nm.

## Methods

### Preparation of ZnS quantum dot solid

The ZnS quantum dots solid were prepared by a modified simple chemical route^43,67,68^. Zn(CH_3_CO_2_)_2_·2H_2_O.and Na_2_S (purchased from Sigma Aldrich) were dissolved separately in double-distilled water. At first, freshly prepared 5 mmol of Zn (OAc_2_) solution was kept in magnetic stirrer. Then varying 50 to 300 µl of 3-mercatopropionic acid (MPA) was added into the above main precursor solution, and the pH of the solution was increased to 9 to 11 by adding NaOH solution. After that, freshly prepared 3 mmole of Na_2_S solution was added slowly drop wise into the above solution to get MPA capped ZnS QD. The mixture is loaded in the round bottom flask under an inert atmosphere with the optimized condition. The reaction flask was maintained at 90 °C with constant stirring, samples were collected at various time intervals (6, 12, 24 and 48 hrs). The white colored colloidal solution was obtained which indicated the formation of ZnS nanocrystal. The sample was washed in (DDW) to remove the unreacted compounds. Further, they were subjected to centrifuge at 5000 rpm for 10 minutes to settle down the particles and then the product was dried at 60 °C under vacuum condition.

### Photocurrent device fabrication

The photocurrent device structure planned for the photocurrent measurement was FTO/TiO_2_/(photosensitizer)/MoO_3_/Ag. Different layers of the device were deposited on the pre-cleaned FTO coated glass slides. TiO_2_ coating (active area 1 × 1 cm^2^) was obtained by using titania paste (purchased from Sigma Aldrich) on FTO substrate by doctor blade technique^69,70^ and the sample was annealed at 450 °C for 6 hrs. The photosensitizer (QD) was deposited by drop cast/spin coating method over the TiO_2_ films and dried at ambient temperature for 6 hrs. The MoO_3_ (hole transport layer) and Ag top electrodes was deposited (electrode area (0.5×0.5 cm^2^) by using thermal evaporation method. The constructed devices were subjected to photocurrent measurements.

### Characterization

HRTEM analysis was carried out using a JEOL JEM-2100 at an operating voltage of 200 kV. UV-Vis absorption measurements were carried out by using an Agilent CARY 60 spectrophotometer. PL excitation and emission measurements were carried out using a Horiba Jobin Yvon fluoromax-4 spectrofluorimeter. The PL lifetime measurements were carried out by using an IBH time-correlated single photon counting (TCSPC) system. The samples were characterized by RAMAN measurements using a Horiba LABRAM HR excited by a 514-nm laser. Photocurrent measurement was carried by using a 300 Watt xenon lamp as an illumination source and a source measuring unit (Agilent B2912A) was used to record the photocurrent values. The photodetector measurements were carried using a UV light source illumination with λ = 390 nm and 8 µW cm^2^ power.

## Supplementary information

Supplementary Information
