# Supplementary material for: Highly Responsive Ultraviolet Sensor Based on ZnS Quantum Dot Solid with Enhanced Photocurrent
Source: Sci Rep. 2019 Dec 10;9:18704. doi: 10.1038/s41598-019-55097-8 (PMC6904578; doi:10.1038/s41598-019-55097-8)
Supplement: Supplementary file 1 — Supplementary Information [file 41598_2019_55097_MOESM1_ESM.docx]

**Supplementary information for**

**Highly Responsive Ultraviolet Sensor Based on ZnS Quantum Dot Solid with Enhanced Photocurrent**

Sellan Premkumar*^1,3^ Devaraj Nataraj*^1,2^ Ganapathi Bharathi^1^ Subramaniam Ramya^1^ and T. Daniel Thangadurai ^4^

1. Quantum Materials and Devices Laboratory, Department of Physics, Bharathiar University, Coimbatore, Tamil Nadu 641046, India.

2. UGC-CPEPA Centre for Advanced Studies in Physics for the development of Solar Energy Materials and Devices, Department of Physics, Bharathiar University, Coimbatore, Tamil Nadu 641046, India.

3. School of Chemistry and Chemical Engineering, Tianjin Polytechnic University, Tianjin 300387, China, Tianjin Key Laboratory of Green Chemistry and Process Engineering, and School of Material Science and Engineering, Tianjin Polytechnic University, Tianjin 300387, China.

4. Department of Nanoscience and Technology, Sri Ramakrishna Engineering College, Coimbatore, Tamil Nadu 641046, India.

**Corresponding Authors E-mail:** [**de.natraj2011@gmail.com**](mailto:de.natraj2011@gmail.com)**,** [**sivajiisro@gmail.com**](mailto:sivajiisro@gmail.com)


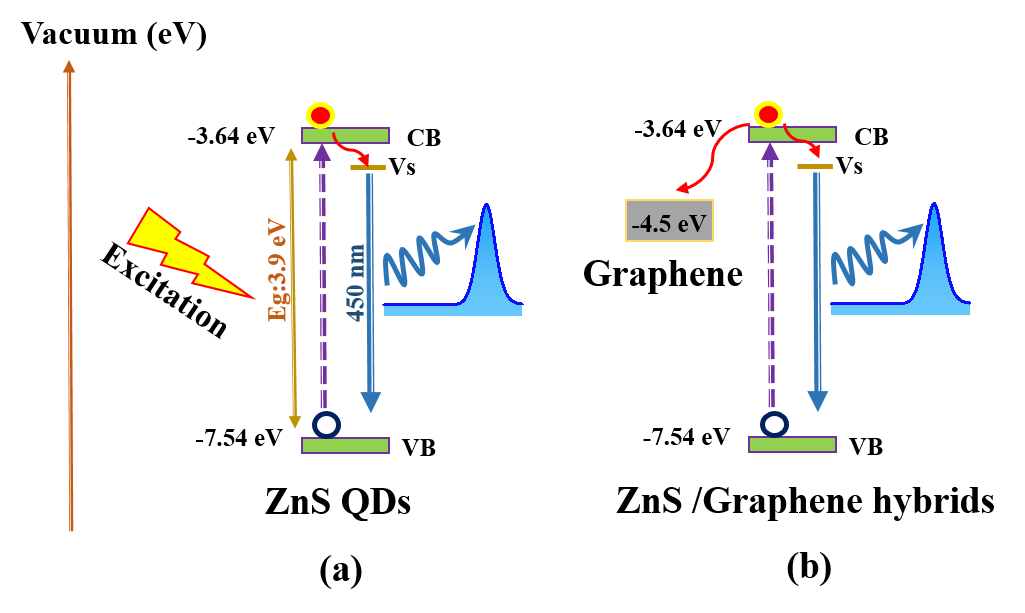


**Figure S1.** Schematic representation of (a) ZnS QD and (b) ZnS QD with graphene hybrids energy band alignment.


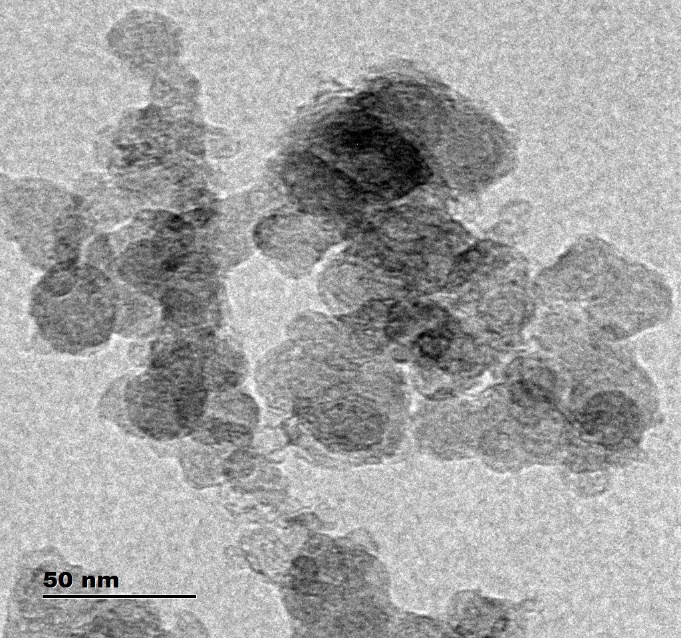

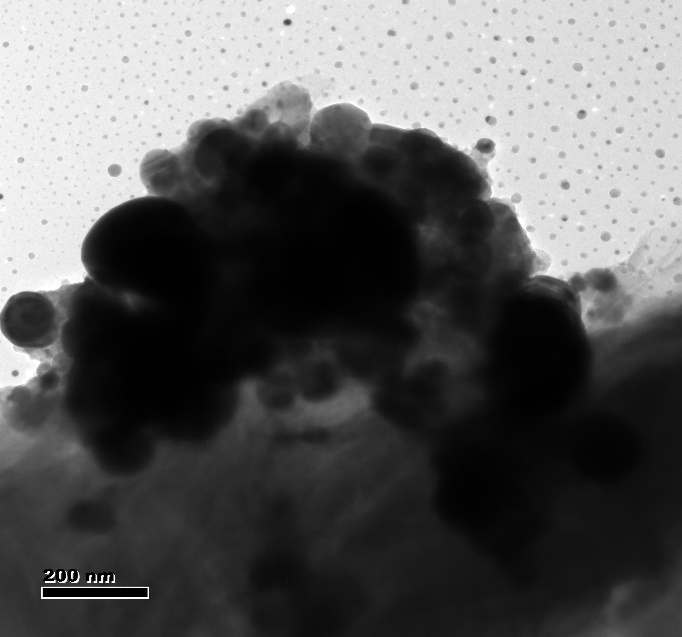


**Figure S2**. HRTEM images of ZnS quantum dot prepared at 48 hrs reaction time (left) low magnification image , and (righ) Higher resolution TEM image- particles combine to form a bigger size particles.

**FTIR analysis**

**Figure S3.** FTIR Spectra of Pure ZnS QDs (top) with Graphene hybrids (bottom) sample prepared at different reaction time intervals.

The FTIR spectra of MPA capped ZnS QDs samples recorded in the range of 4000-550 cm^-1 ­­‑^are shown in Fig. S3. The symmetric stretching vibration bands observed at 1393 cm^-1^ and 1400 cm^-1^ corresponds to OH deformation vibration of ternary C-OH.[^1^](#_ENREF_1)^,^[^2^](#_ENREF_2) The peak at 1649 cm^-1^ was observed due to C=O stretching vibration. The obtained result shows the fact that upon the interaction of MPA with ZnS, the typical (S-H) stretching vibration band at 2550-2678 cm^-1^  got disappeared[^3^](#_ENREF_3). This observation confirms the formation of ZnS. Fig. S3 shows the FTIR spectra of ZnS QDs/Graphene hybrids samples where a major band present in graphene oxide 829 cm^-1^ is due to the stretching of C-H out of the plane edge, the peak at 1040 cm^-1^ is attributed for C-O stretching vibration and the corresponding peak at 1642 cm^-1^ is due to C=O stretching vibration[^4-8^](#_ENREF_4). The presence of different type functionalities in graphene hybrid sample prepared at 24 hrs was confirmed and also decreased intensity confirms the formation of graphene presence in the ZnS hybrids samples.


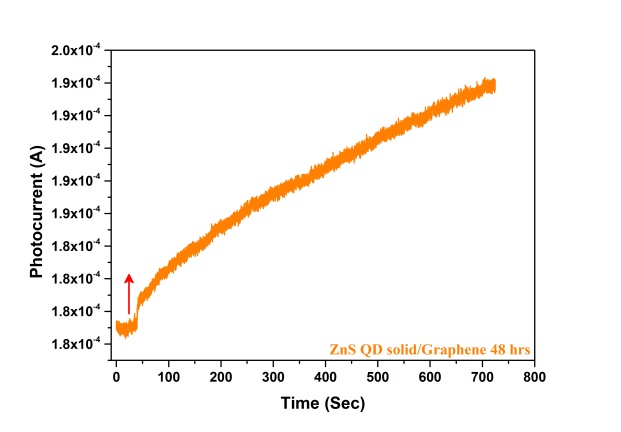

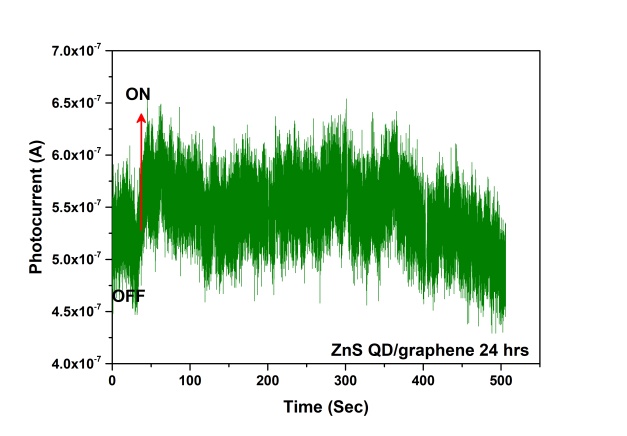

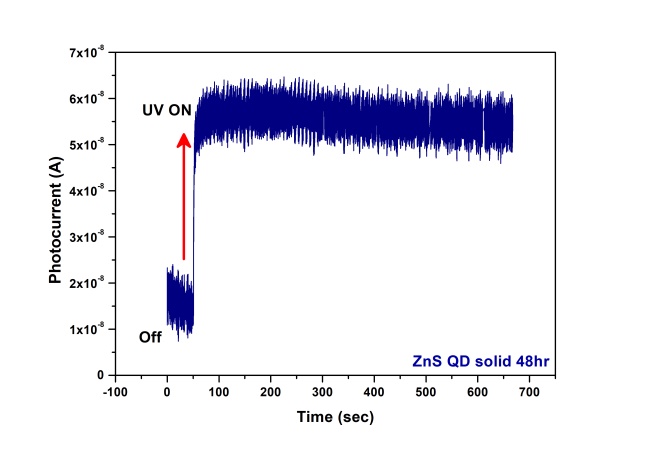

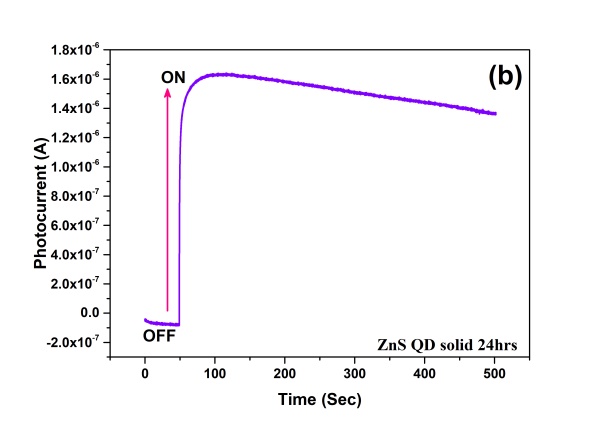


**Figure S4**. The stability curve of the prepared UV photodetector device under continuous UV light (390 nm) illumination for (a) ZnS QD solid 24 hrs, (b) ZnS QD solid 48hrs, (c) ZnS QD solid/graphene hybrids 24hrs and (d) ZnS QD solid/graphene hybrids 48hrs.

The stability of the constructed devices was examined by continuous illumination and the photocurrent curves are presented in Fig S4. In the case of device which is suitable for practical applications (bare ZnS QD solid device 24 hr), there is only a slight decrease in the maximum photocurrent after a continuous illumination exceeding 500 seconds. This device can retain the maximum photocurrent in the range 1.6 μA, up to 500sec. The other devices also displayed better stability when continuous illumination as shown in Fig.S4.

**References**

1 Barth, A. Infrared spectroscopy of proteins. *Biochimica et Biophysica Acta (BBA) - Bioenergetics* **1767**, 1073-1101, doi:https://doi.org/10.1016/j.bbabio.2007.06.004 (2007).

2 Marrone, M. *et al.* A Fourier Transform Infrared (FTIR) Study of the Reaction of Triethoxysilane (TES) and Bis[3-triethoxysilylpropyl]tetrasulfane (TESPT) with the Surface of Amorphous Silica. *The Journal of Physical Chemistry B* **108**, 3563-3572, doi:10.1021/jp036148x (2004).

3 Premkumar, S., Nataraj, D., Bharathi, G., Khyzhun, O. Y. & Thangadurai, T. D. Interfacial Chemistry-Modified QD-Coupled CdTe Solid Nanowire and Its Hybrid with Graphene Quantum Dots for Enhanced Photocurrent Properties. *ChemistrySelect* **2**, 10771-10781, doi:10.1002/slct.201702352 (2017).

4 Rattana *et al.* Preparation and characterization of graphene oxide nanosheets. *Procedia Engineering* **32**, 759-764, doi:https://doi.org/10.1016/j.proeng.2012.02.009 (2012).

5 Lewis, P. D. *et al.* Evaluation of FTIR spectroscopy as a diagnostic tool for lung cancer using sputum. *BMC Cancer* **10**, 640-640, doi:10.1186/1471-2407-10-640 (2010).

6 Suryawanshi, A. *et al.* Large scale synthesis of graphene quantum dots (GQDs) from waste biomass and their use as an efficient and selective photoluminescence on–off–on probe for Ag+ ions. *Nanoscale* **6**, 11664-11670, doi:10.1039/c4nr02494j (2014).

7 Miyazawa, T., Shimanouchi, T. & Mizushima, S. i. Normal Vibrations of N‐Methylacetamide. *The Journal of Chemical Physics* **29**, 611-616, doi:10.1063/1.1744547 (1958).

8 Galande, C. *et al.* Quasi-Molecular Fluorescence from Graphene Oxide. *Scientific Reports* **1**, 85, doi:10.1038/srep00085

https://[www.nature.com/articles/srep00085#supplementary-information](http://www.nature.com/articles/srep00085#supplementary-information) (2011).
